# A Comparison of SCC Behaviors for Two States of Fe_85_Ga_15_ Alloys in a 3.5 wt % NaCl Solution

**DOI:** 10.3390/ma13112536

**Published:** 2020-06-03

**Authors:** Suli Zhao, Junliang He, Xuewei Zhang, Jinxu Li

**Affiliations:** Corrosion and Protection Center, Institute for Advanced Materials and Technology, University of Science and Technology Beijing, Beijing 100083, China; zhaosl20180123@gmail.com (S.Z.); h593890945@gmail.com (J.H.); B20190582@xs.ustb.edu.cn (X.Z.)

**Keywords:** Fe-Ga alloy, SCC, normalized threshold stress, anodic dissolution

## Abstract

The new magnetostrictive material Fe-Ga alloy has drawn considerable attention due to its excellent performance. The microstructure of the Fe-Ga alloy, which varies with the state of preparation or processing, not only affects the magnetostriction but also the mechanical service stability, i.e., the stress corrosion cracking (SCC) property. In this paper, we report a comparison of the SCC behaviors for two states of Fe_85_Ga_15_ alloys by using constant load experiments and the electrochemical method. Results showed that the hot-rolled Fe_85_Ga_15_ alloy exhibited better mechanical properties compared to the as-cast Fe_85_Ga_15_ alloy. SCC was found in both Fe_85_Ga_15_ alloys in a 3.5 wt % NaCl solution, and the as-cast alloy was more sensitive to SCC. The normalized thresholds of SCC for the as-cast and hot-rolled Fe_85_Ga_15_ alloys are 0.34 and 0.81, respectively. Furthermore, results also indicated that the SCC mechanism is an anodic dissolution for the Fe_85_Ga_15_ alloy.

## 1. Introduction

Magnetostrictive Fe-Ga alloys with excellent comprehensive properties have been given more attention recently. The increase of the lattice constant, due to Ga substituted for Fe in the common bcc structure [1] causes lattice distortion. In addition, a gallium-rich atomic cluster and a small amount of B_2_, DO_3_, and B_2_-like ordered phase, etc., might exist in these alloys [2,3,4]. These features enhanced the saturation magnetostriction coefficient of Fe-Ga alloys enormously to 200–400 ppm [5,6,7,8,9]. Corrosion and stress corrosion cracking (SCC) are sensitive to defects in crystals, such as a grain boundary, dislocation, and segregation. Therefore, the presence of the lattice distortion and microstructure mentioned above will have a significant impact on the corrosion and SCC of alloys in comparison with α-Fe, even if the percentage is small in the solid solution. On the other hand, the Fe-Ga alloy is a new type of magnetostrictive material after the TbDyFe and NiMnGa alloys. It offers a higher potential application value due to its more excellent mechanical properties [8,9,10], and easier fabrication procedures compared to the former magnetostrictive materials [10,11]. The combination of these properties in the Fe-Ga alloy makes it attractive for various applications that include new underwater acoustic transducers, sonar detectors, actuators, vibration power generators and microphones [12]. Hence, the investigation of the performance in possible service environments is essential to the application of Fe-Ga alloys.

Fe-Ga alloys are a new kind of magnetostrictive material with good mechanical properties and a large magnetostrictive coefficient compared to traditional magnetostrictive materials (Fe, Co, Ni, and Fe-Al) and rare earth magnetostrictive materials (TbDyFe), and they are considered as the new generation of giant magnetostrictive material. It seems that the new magnetostrictive material possesses a higher environment fracture sensitivity either NiMnGa or TbDyFe [13,14,15,16,17]. Gebert [13] investigated the corrosion behavior of polycrystalline Ni_2_MnGa, and found that the alloy exhibited a higher reactivity in acidic solutions and a lower corrosion rate in alkaline solutions. Shen et al. [14] studied the SCC of the Ni_2_MnGa alloy in deionized water and moist atmosphere. Their results proved that the cracks could propagate in water and moist atmosphere. The fracture toughness decreased drastically from [15] when the hydrogen concentration in the sample reached 19.9 ppm, suggesting the SCC sensitivity is higher after hydrogen charging. Sachdeva [16] studied the corrosion behavior of (TbDy)Fe_2_, and the results showed that corrosion rates were higher in 3.5 wt % NaCl solutions than in 0.01 N Na_2_SO_4_ solutions. Niu [17] showed that an unloaded indentation crack could delay propagation in humid air. In conclusion, serious corrosion and SCC could occur in NaCl solutions for magnetostrictive materials such as TbDyFe and NiMnGa.

Numerous studies have been conducted on the corrosion and SCC phenomenon of pure iron and low alloy steel. SCC would not occur in an NaCl solution for the ingot iron. However, SCC would occur if a certain concentration of hydrogen existed in the sample, and the plasticity loss induced by hydrogen reached at least 65% when the content of hydrogen was 5 ppm [18]. Normally, SCC will not occur in an NaCl solution for low alloy steels such as HY100 and S355N [19,20].

For an Fe-Ga alloy, whose chemical composition stability lies between that of rare earth magnetostrictive materials and that of pure iron, corrosion in an NaCl solution has been reported. Jayaraman [21] studied the corrosion behavior of Fe-Ga alloys in a 3.5 wt % NaCl solution, a 0.1 M HCl solution, and a 0.1 M NaOH solution, and drew the conclusion that the corrosion rate was highest in an 0.1 M HCl solution and lowest in an 0.1 M NaOH solution. The same author [22] also studied the impact of the magnetic field on the corrosion behavior of Fe-Ga alloys in an NaCl solution and an HCl solution. Ramanathan [23] studied the influence on the mechanical behavior of the Fe-Ga alloy with and without a magnetic field and hydrogen. Our previous work [24] reported the effect of a magnetic field on the SCC of the Fe_83_Ga_17_ alloy, and the results showed that SCC was enhanced when a tensile magnetic field was applied, and inhibited when an in-plane or perpendicular magnetic field was applied. He [25] preliminarily showed that anodic dissolution is the SCC mechanism of the Fe_85_Ga_15_ alloy.

Up to now, extensive research has been conducted on the Fe-Ga alloy, and most of it has focused on the material preparation, microstructure, and magnetostriction [3,4,5,6,7,8,9,10]. However, hardly any research has focused on the SCC properties of the Fe-Ga alloy, despite several reports on the corrosion behavior [21,22,23,24,25]. It is important to study the physical properties of the Fe-Ga alloy as a functional device; however, the mechanical stability in preparation and service processes should not be ignored either. Environmental factors could affect a mechanical and structural stability in service, such as SCC [26,27,28,29,30,31]. In addition, the microstructure in the material could induce changes in the electrochemical behavior in certain environments [32,33,34,35]. The preparation and processing of the material seriously affect the mechanical and SCC performance, and thus the service (such as the microstructure and mechanical properties) and functional stability. In this paper, the SCC and electrochemical corrosion of as-cast and hot-rolled Fe_85_Ga_15_ alloys in a 3.5 wt % NaCl solution are studied, to provide a reference for preparing functional device materials and determining which Fe_85_Ga_15_ alloy state is more suitable for promotion and application.

## 2. Materials and Methods 

Two states of Fe_85_Ga_15_ alloys—as-cast and hot-rolled—were used in this work. The Fe_85_Ga_15_ alloy in cast was prepared by the following process: Fe_85_Ga_15_ alloy ingots of approximately 1000 g were prepared from pure Fe (99.95 wt % purity), pure Ga (99.995 wt % purity), and a compound according to the target composition. Although Ga has a higher vapor pressure than Fe under the melting temperature, the Ga element burnt loss could be neglected through carefully controlling the melting process. The cylindrical master alloy with a diameter of 10 mm and a length of 100 mm was obtained by induction melting under an argon atmosphere (Z0-001). After homogenizing treatment in an argon atmosphere at 1100 °C, the alloy was cooled from 1100 to 730 °C in a furnace and preserved for 3 h at 730 °C, and then cooled to room temperature in the furnace. The hot-rolled Fe_85_Ga_15_ alloy was prepared by the following process: The melting process was the same as above. Then the cast ingot was annealed at 1150 °C for 30 min, followed upsetting and extension forging by air hammer (C41-750B) for 5 passed to a thickness of 20 mm. The ingot was then hot-rolled to a 1.5 mm thickness sheet. All the samples of both alloys were annealed at 600 °C for 2 h in an argon atmosphere and then air-cooled to room temperature. The microstructure and fracture surfaces of the samples tested were examined using an optical microscope and scanning electron microscopy.

The saturation magnetostriction was measured by resistance strain gauges (JDM-30, Beijing, China) at room temperature. The dimensions of samples were 30 × 15 × 1.3 mm^3^, and the samples of hot-rolled alloy were located along the rolling direction. The saturation magnetostriction are calculated by (3/2)λs = λparallel − λperpendicular.

The microstructure was examined by optical metallographic microscope (OLYMPUS BX60, Olympus Corporation, Tokyo, Japan). The samples (10 × 15 × 1.3 mm^3^) were sanded by sandpaper from 400# to 5000#, and then polished with 0.5 µm diamond pastes. Then, the samples were eroded with 4% alcohol nitrate solution.

The tensile tests and constant load tests were performed on the plate tensile samples with the dimensions of 15 × 3 × 1.3 mm^3^, whose axis parallel to the rolling direction of the alloy. The rate of slow strain rate tension (SSRT) was 5 × 10^−7^ s^−1^. All the samples were progressively ground with sandpaper up to 2000#, and samples were then ultrasonically cleaned in acetone and alcohol before the tests.

The smooth tensile samples were put into the corrosive medium under constant stress. SCC was generated after an incubation period until rupture. The delayed fracture time and the threshold stress σ_SCC_ were recorded based on the stress–fracture time curves:

σ_SCC_ = 1/2 (σ_f_ + σ_n_) (1)

where σ_f_ is the minimum stress for delayed fracture to occur during a defined time t_c_ (at least 300 h). σ_n_ is the maximum stress for not inducing fracture during a defined time. In order to ensure that the relative error between the experimental value and the true value of σ_SCC_ is less than 5%, the relation between σ_f_ and σ_n_ is given:

σ_f_ − σ_n_ ≤ 0.1 σ_SCC_ (2)

Potentiodynamic polarization measurements were tested in a three-electrode system where a Pt plate was utilized as the counter electrode, a saturated calomel electrode was the reference electrode, and a sample was the working electrode. The electrochemical tests were performed with an electrochemical workstation (CHI660E, CH Instruments, Austin, TX, USA). The polarization tests were conducted within a potential range from −300 mV vs. open circuit potential (OCP) to 300 mV vs. OCP at a scanning rate of 1 mV/s.

The X-ray diffraction (XRD; DMAX-RB, Rigaku, Tokyo, Japan) samples underwent continuous scanning at 0.2°/min in the 2θ range of 20° to 90°. The fracture surface and surface morphology were observed by scanning electron microscopy (SEM, $3400N, Hitachi, Japan). The element distribution were analyzed by energy dispersive spectra (EDS), which attached to the SEM ($3400N).

## 3. Results

### 3.1. Characterization of the Fe_85_Ga_15_ Alloys

**Figure 1:**
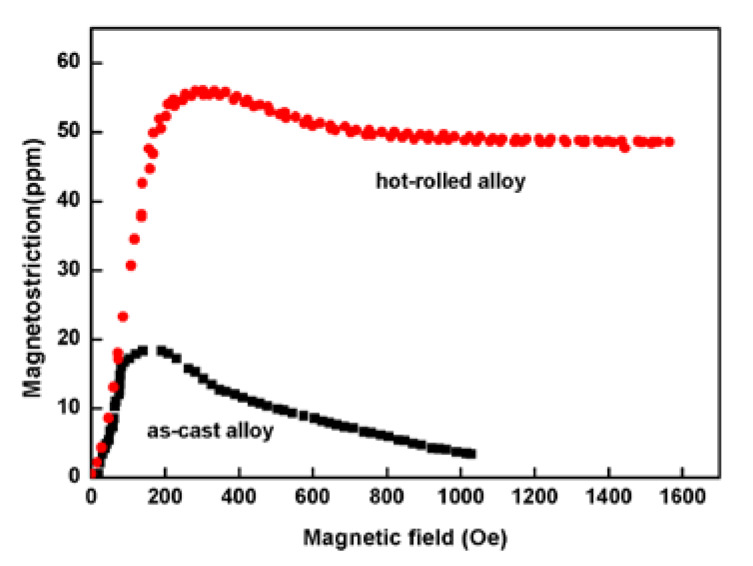
Magnetostriction for the two states of Fe_85_Ga_15_ alloys.

**Figure 2:**
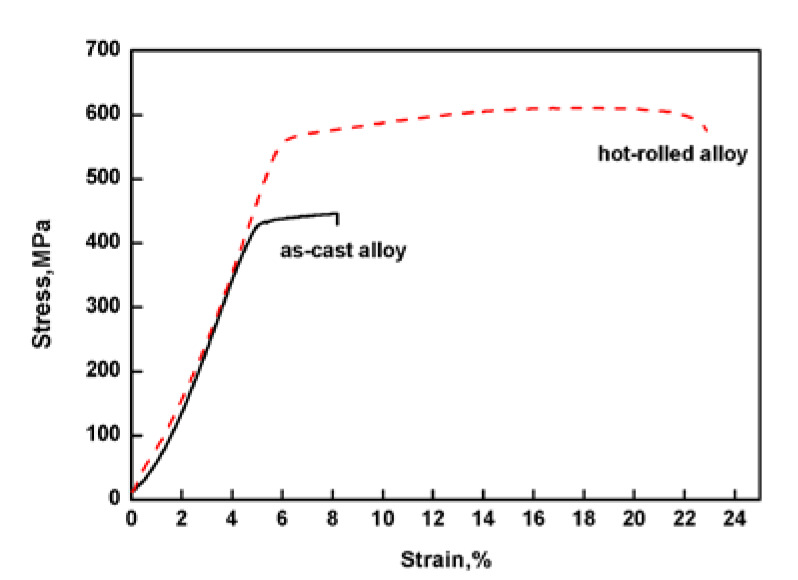
Stress–strain curves of the two alloys tested in air.

Figure 1 shows that the saturation magnetostriction of the as-cast and hot-rolled was 18 ppm and 55 ppm, respectively, i.e. the hot-rolled alloy exhibits a higher saturation magnetostriction. The tensile strength values (σ_b_) of the as-cast Fe_85_Ga_15_ alloy and hot-rolled Fe_85_Ga_15_ alloy were 435 MPa and 605 MPa, respectively, and the elongation values (δ_b_) of the two alloys were 3.6% and 16.8%, respectively, as shown in Figure 2. Apparently, the strength and plasticity are significantly improved after hot rolling.

**Figure 3:**
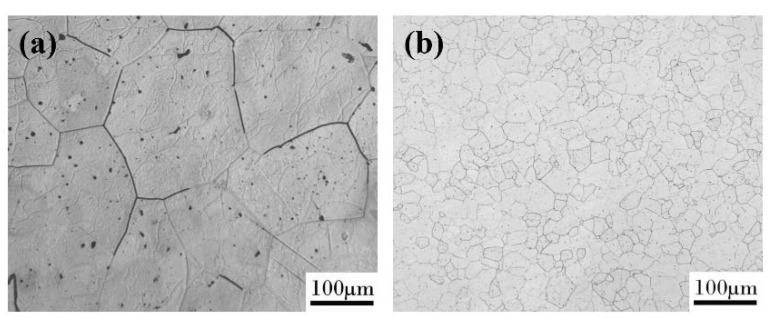
Surface morphology observed in an optical microscope: (**a**) as-cast alloy; (**b**) hot-rolled alloy.

**Figure 4:**
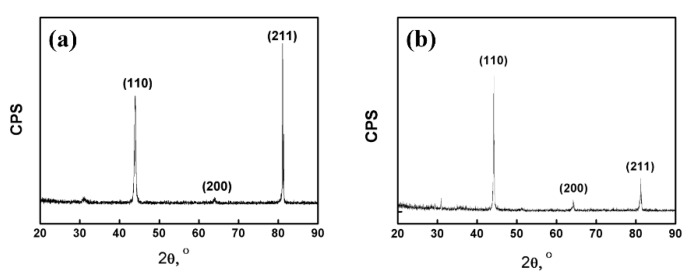
X-ray diffraction: (**a**) as-cast alloy; (**b**) hot-rolled alloy.

The metallographic structure was shown in Figure 3a,b. It can be seen that the microstructure of the as-cast alloy was coarse. The grain boundary grooves of the as-cast Fe_85_Ga_15_ alloy are obviously visible, and there are sub-grain boundaries in the interior of grains. The hot-rolled alloy exhibits a dynamic recrystallized and a fine equiaxed grain structure, and the surface is smoother than the as-cast alloy. The precipitates distribute more homogeneously in the grains and sub-grain boundaries in the two alloys, and a few precipitates were located in the interior of the grains. The XRD spectrum of the two alloys is shown in Figure 4a,b, indicating that the stable phase of the two alloys at room temperature is single-phase ferrite, whose structure is similar to α−Fe.

### 3.2. The SCC Threshold Stress σ_SCC_

**Figure 5:**
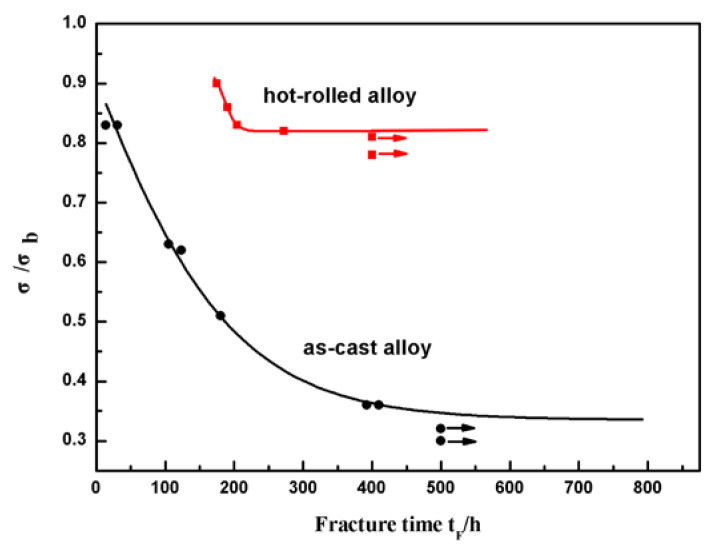
Fracture time t_F_ vs. normalized stress σ/σ_b_ for two states of Fe_85_Ga_15_ alloys in the 3.5 wt % NaCl solution.

Figure 5 shows the normalized stress σ/σ_b_ at various fracture times for the as-cast Fe_85_Ga_15_ alloy and hot-rolled Fe_85_Ga_15_ alloy in a 3.5 wt % NaCl solution. The result shows that, under the open circuit potential, the rupture time of SCC increases as the applied stress decreases. It is evident that the normalized stress σ/σ_b_ at various fracture times for the hot-rolled Fe_85_Ga_15_ alloy shifted to the top right in comparison to that observed for the as-cast Fe_85_Ga_15_ alloy in the 3.5 wt % NaCl solution. According to Equation (1), the normalized threshold of SCC is σ_SCC_/σ_b_ = 0.34, and the corresponding threshold stress (σ_SCC_) is 148 MPa for the as-cast Fe_85_Ga_15_ alloy. The normalized threshold of SCC is σ_SCC_/σ_b_ = 0.81, and the corresponding threshold stress (σ_SCC_) is 490 MPa for the hot-rolled Fe_85_Ga_15_ alloy. In contrast to the hot-rolled alloy, the as-cast alloy has a much higher SCC sensitivity. That is, not only are the mechanical properties hugely improved, but the SCC sensitivity is also reduced after hot rolling [36,37,38]. The above result shows that the grains were refined after hot rolling, and the excellent corrosion resistance of the hot-rolled alloy therefore originates from the grain refinement. Previous work showed that SCC tended to take place at the site of large grain size, and SCC resistance was increased by grain refinement [39]. 

### 3.3. SEM Fractography

**Figure 6:**
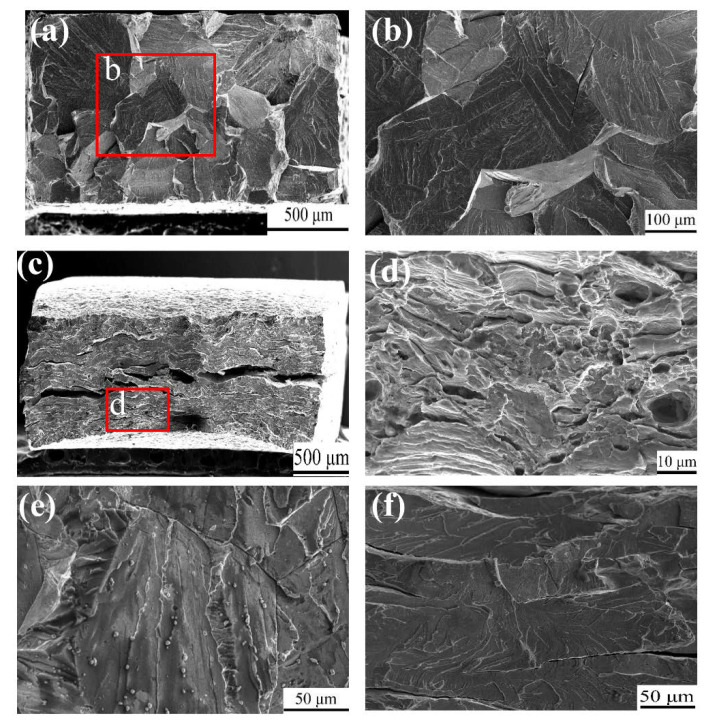
SEM fracture surface tensiled in air: (**a**) as-cast; (**c**) hot-rolled; (**b**,**d**) magnified of the parts of red rectangles; tensiled in a 3.5 wt % NaCl solution with 5 × 10^−7^ s^−1^: (**e**) as-cast; (**f**) hot-rolled.

The fracture surface images of the two states of Fe_85_Ga_15_ alloys tensiled in air are shown in Figure 6a–d. Figure 6a shows an almost flat surface of the sample cut from the as-cast Fe_85_Ga_15_ alloy, and the higher magnification of area of the red rectangle is shown in Figure 6b, which shows a typical cleavage fracture. This is because of the brittleness of the as-cast alloy itself, and the elongation value of the as-cast alloy is only 3.6%. Figure 6c shows that the fracture surface of the sample cut from hot-rolled Fe_85_Ga_15_ alloy is found with both cleavage fracture as well as some ductile fracture, e.g., an obvious necking phenomenon. The ductile fracture surface is observed in the vicinity of the surface of the sample, as indicated by the red rectangle. The higher magnification of the red rectangle area is shown in Figure 6d. Multiple steps were observed in the fracture surface, suggesting that there was some plastic deformation during the fracturing process. Figure 6e,f shows fracture surface images of the two alloys tensiled in the 3.5 wt % NaCl solution at a rate of 5 × 10^−7^ s^−1^. In both samples, the fracture morphology was a cleavage fracture. Similar fracture features can be observed in samples cut from the as-cast alloy tested in air and in the 3.5 wt % NaCl solution. In contrast, no ductile feature can be observed on the sample cut from the hot-rolled alloy in the 3.5 wt % NaCl solution compared to the sample tested in air.

### 3.4. Influence of Polarization Potential on Constant Load SCC

**Figure 7:**
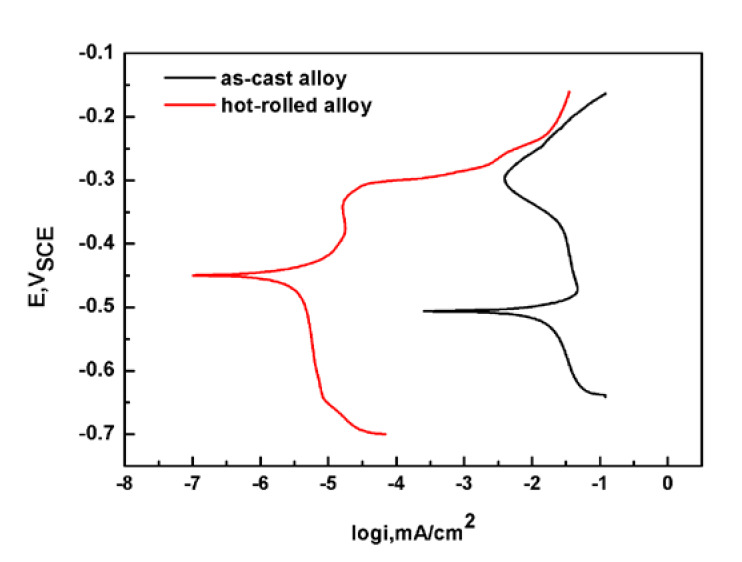
Polarization curves of the two states of Fe_85_Ga_15_ alloys in the 3.5 wt % NaCl solution.

Polarization curves of the as-cast Fe_85_Ga_15_ and hot-rolled Fe_85_Ga_15_ alloy in the 3.5 wt % NaCl solution are shown in Figure 7, which were performed from (OCP −300 mV) to (OCP +300 mV). Both of them show a trend from active to passive, and neither of them were stable. It can be seen that there is a substantial difference between the two polarization curves. The curve of the hot-rolled Fe_85_Ga_15_ alloy is left of the curve of the as-cast Fe_85_Ga_15_ alloy. These results show that the corrosion potential values of the as-cast Fe_85_Ga_15_ and hot-rolled Fe_85_Ga_15_ alloys were −510 and −450 mV, and the corrosion current density values were 4 × 10^−3^ and 5 × 10^−6^ A/cm^2^, respectively. According to the electrochemistry theory, corrosion resistance of the hot-rolled Fe_85_Ga_15_ alloy is better than the as-cast Fe_85_Ga_15_ alloy. Previous studies have shown that crystal defects can affect material corrosion [40,41,42]. Grain size has also proven to be a key factor that affects the corrosion property. Miyamoto et al. [43] studied the corrosion of coarse grain copper and ultra-fine grain copper, and the results showed that ultra-fine grain copper had a lower corrosion current compared to coarse grain copper. In comparison to ultra-fine grain copper, grain boundary grooves were more obviously visible, and the grain boundaries of ultra-fine grain copper were less attacked [43]. Wang showed that nano-crystalline of 304SS exhibited higher resistance to corrosion compared to coarse-grained 304SS [44]. Afshari demonstrated that charge transfer impedance increased as the grain size decreased [45]. In our case, the grain size difference between the hot-rolled and as-cast Fe-Ga alloys is also substantial. Grains of the hot-rolled Fe_85_Ga_15_ alloy are evidently finer and more uniform, while the grain boundary grooves in the as-cast Fe_85_Ga_15_ alloy are clearly visible, which could be attacked more easily in the 3.5 wt % NaCl solution.

**Figure 8:**
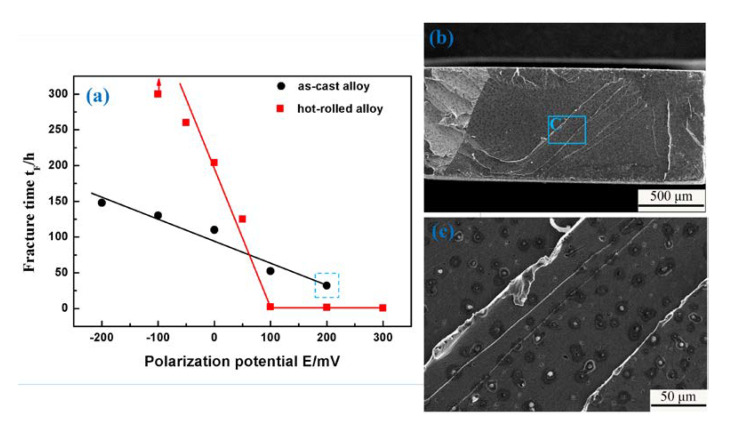
(**a**) Fracture time vs. polarization potential in the 3.5 wt % NaCl solution; (**b**) SEM fracture surface of the data point of blue dashed rectangle in (**a**); (**c**) magnified of the part of blue rectangle.

The relationship between fracture time and polarization potential in the 3.5 wt % NaCl solution is shown in Figure 8a. The tests were conducted under a constant load stress of 300 MPa and 502 MPa for the as-cast and hot-rolled alloys, respectively. It can be seen that the time to fracture increased with the increment of the absolute value of cathodic polarization potential in both samples. When the value of cathodic polarization is −100 mV, no fracture was observed in the hot-rolled alloy sample, indicating that SCC is inhibited by cathodic polarization. On the contrary, for both kinds of samples, the time to fracture decreased with the increasing anodic polarization potential, even the fracture time of the hot-rolled sample is close to zero. This means that the SCC is promoted by the anodic polarization. These results also suggest that the hot-rolled alloy could service safely for a long time under cathodic polarization, while the as-cast alloy cannot. Moreover, this alloy is generally not used under anodic polarization. Figure 8b shows the fracture surface morphology of the as-cast Fe_85_Ga_15_ alloy test under the constant load stress of 300 MPa and anodic polarization value of 200 mV, which corresponds to the data point of the blue dashed rectangle in Figure 8a. It is observed that the fracture is more flat, and the higher magnification of area of the blue rectangle is shown in Figure 8c, which shows obvious corrosion pits.

**Figure 9:**
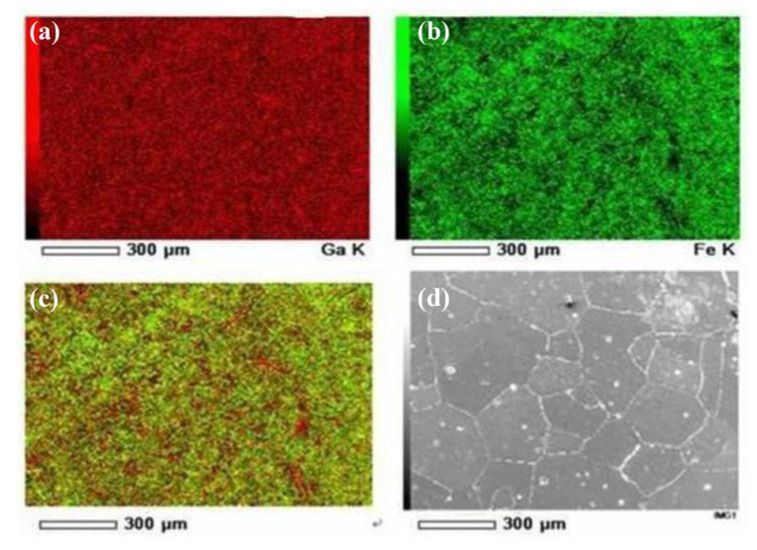
The surface morphology and EDS analysis of the as-cast Fe_85_Ga_15_ alloy after polishing. (**a**) Ga; (**b**) Fe; (**c**) overlap of Ga and Fe; (**d**) surface morphology.

The element distribution of the as-cast Fe_85_Ga_15_ alloy after polishing was analyzed by EDS, as shown in Figure 9. Figure 9a,b show the distribution of Ga and Fe on the surface, respectively. Figure 9c shows all elements distribution on the surface, because there is only two elements, i.e. the distribution of Ga and Fe. It was observed that the segregation of Ga occurred on the as-cast alloy grain boundaries. This could be attributed to the energy difference of distortion that was generated in the grain and grain boundary, due to the size discrepancy between solute and parent atoms. Our previous work showed that an intergranular crack is the origin of stress corrosion crack for the Fe-Ga alloy [24]. He [25] showed that there are some pits that distribute densely on the sample’s surface of the as-cast Fe_85_Ga_15_ alloy after mechanical polishing, and most of them present in the vicinity of the grain boundary. The EDS results show that the content of Ga in the precipitate was higher than that in the matrix [25]. Therefore, a homogeneous distribution is important in the material, and the segregation of Ga should be avoided, in order to prevent the origin of the deterioration.

## 4. Discussion

In this work, it was found that delayed fracture can occur in both as-cast and hot-rolled Fe_85_Ga_15_ alloys under constant stress far below their tensile strength in a 3.5 wt % NaCl solution, which is to say that SCC can occur in Fe_85_Ga_15_ alloys. This means that, after Ga substituted for Fe forming solid solution, the SCC sensitivity in a 3.5 wt % NaCl solution significantly increases for an Fe_85_Ga_15_ alloy compared to pure iron and low alloy steel. It is not surprising that little distinction was observed between the macroscopic fracture surfaces tested in air and tested in the 3.5 wt % NaCl solution of the as-cast Fe_85_Ga_15_ alloy because of its brittle nature.

The standard electrode potential of iron is −0.440 V, which is obtained on the basis of electrode reaction equation Fe = Fe^2+^ + 2e at room temperature, and the standard electrode potential of gallium is −0.53 V, which is obtained on the basis of electrode reaction equation Ga = Ga^3+^ + 3e at room temperature. In the Fe-Ga alloy, gallium acts as the anode, and will dissolve in a 3.5 wt % NaCl solution relative, as a gallium-rich zone of the Fe-Ga alloy has a lower potential. When the Fe-Ga alloy is corroded, the anodic reaction equation is described as follows:

Ga→Ga^3+^ + 3e or Fe→Fe^2+^ + 2e (3)

The cathodic reaction is as follows:

2H_2_O→2H^+^ + 2OH^-^ and 2H^+^ + 2e→2H. (4)

If hydrogen atoms generated from cathodic reaction could diffuse into the sample and control the crack nucleation and propagation process, then it could be recognized as a hydrogen-induced cracking of stress corrosion. For a low intensity steel and iron alloy, the threshold hydrogen concentration to cracking is far beyond that of high strength steels. Therefore, the hydrogen concentration for steel to corrosion in neutral aqueous solution is below the threshold concentration of hydrogen-induced cracking, so the crack nucleation and propagation of stress corrosion could only be feasible through anodic dissolution. Our results showed that the SCC of the Fe_85_Ga_15_ alloy mechanism is also anodic dissolution, as shown in Figure 8, as cathodic polarization could promote hydrogen evolution and improve the hydrogen concentration in the samples. The experiments showed that the time to fracture increased with the increase in the absolute value of cathodic polarization potential in both alloys, and anodic polarization reduced the fracture time, which results from the promotion of the anodic polarization on anodic dissolution. 

As a functional material, the Fe-Ga alloy should not only possess excellent mechanical properties, but also exhibit good SCC resistance during the service process. The mechanical stability further influences the stability of function. In this work, the mechanical property and constant load tests were conducted on two Fe_85_Ga_15_ alloys with different microstructures. Results showed that the hot-rolled Fe_85_Ga_15_ alloy has better mechanical properties and SCC resistance. This work shows that the properties of the hot-rolled alloy is better than the as-cast alloy, and we suggest that the hot-rolled Fe-Ga alloy be used in the fabrication of devices.

## 5. Conclusions

The comparison of the SCC behaviors for the as-cast and hot-rolled alloys was investigated. The major results can be summarized as follows:

(1) After hot rolling, the Fe_85_Ga_15_ alloy showed better mechanical properties, the tensile strength value increased by about 170 MPa, and the elongation value increased about 13% compared to that of the as-cast Fe_85_Ga_15_ alloy. Hot rolling refined the grains and improved the uniformity of the Fe_85_Ga_15_ alloy.
(2) SCC of the Fe_85_Ga_15_ alloy could occur in a 3.5 wt % NaCl solution. The normalized threshold of SCC was σ_SCC_/σ_b_ = 0.34 for the as-cast Fe_85_Ga_15_ alloy, and the normalized threshold of SCC is σ_SCC_/σ_b_ = 0.81 for the hot-rolled Fe_85_Ga_15_ alloy. The hot-rolled Fe_85_Ga_15_ alloy shows a higher SCC resistance.
(3) The fracture time is increased under cathodic polarization but decreased under anodic polarization for the two Fe_85_Ga_15_ alloys in the 3.5 wt % NaCl solution. Therefore, the results suggest that SCC mechanism is might controlled by anodic dissolution.

In summary, both the mechanical properties and the degradation of the environment performance, i.e., the SCC performance in service, were affected by the microstructure. The hot-rolled Fe_85_Ga_15_ alloy, compared with the as-cast alloy, had a higher magnetostriction and a better performance in terms of mechanical properties and SCC resistance under electrochemical polarization. This paper provides a reference for the material preparation of Fe-Ga alloys as functional devices.
